# Impaired Cardiac SIRT1 Activity by Carbonyl Stress Contributes to Aging-Related Ischemic Intolerance

**DOI:** 10.1371/journal.pone.0074050

**Published:** 2013-09-10

**Authors:** Chunhu Gu, Yuan Xing, Li Jiang, Mai Chen, Ming Xu, Yue Yin, Chen Li, Zheng Yang, Lu Yu, Heng Ma

**Affiliations:** 1 Department of Cardiovascular Surgery, Xijing Hospital, Fourth Military Medical University, Xi’an, China; 2 Department of Physiology, Tangdu Hospital, Fourth Military Medical University, Xi’an, China; 3 Department of Plastics and Burns, Tangdu Hospital, Fourth Military Medical University, Xi’an, China; 4 Department of Cardiology, Xijing Hospital, Fourth Military Medical University, Xi’an, China; 5 Department of Pathology, Xijing Hospital, Fourth Military Medical University, Xi’an, China; University of Western Ontario, Canada

## Abstract

Reactive aldehydes can initiate protein oxidative damage which may contribute to heart senescence. Sirtuin 1 (SIRT1) is considered to be a potential interventional target for I/R injury management in the elderly. We hypothesized that aldehyde mediated carbonyl stress increases susceptibility of aged hearts to ischemia/reperfusion (I/R) injury, and elucidate the underlying mechanisms with a focus on SIRT1. Male C57BL/6 young (4-6 mo) and aged (22-24 mo) mice were subjected to myocardial I/R. Cardiac aldehyde dehydrogenase (ALDH2), SIRT1 activity and protein carbonyls were assessed. Our data revealed that aged heart exhibited increased endogenous aldehyde/carbonyl stress due to impaired ALDH2 activity concomitant with blunted SIRT1 activity (*P*<0.05). Exogenous toxic aldehydes (4-HNE) exposure in isolated cardiomyocyte verified that aldehyde-induced carbonyl modification on SIRT1 impaired SIRT1 activity leading to worse hypoxia/reoxygenation (H/R) injury, which could all be rescued by Alda-1 (ALDH2 activator) (all *P*<0.05). However, SIRT1 inhibitor blocked the protective effect of Alda-1 on H/R cardiomyocyte. Interestingly, myocardial I/R leads to higher carbonylation but lower activity of SIRT1 in aged hearts than that seen in young hearts (*P*<0.05). The application of Alda-1 significantly reduced the carbonylation on SIRT1 and markedly improved the tolerance to *in vivo* I/R injury in aged hearts, but failed to protect Sirt1^+/−^ knockout mice against myocardial I/R injury. This was verified by Alda-1 treatment improved postischemic contractile function recovery in *ex vivo* perfused aged but not in Sirt1^+/−^ hearts. Thus, aldehyde/carbonyl stress is accelerated in aging heart. These results provide a new insight that impaired cardiac SIRT1 activity by carbonyl stress plays a critical role in the increased susceptibility of aged heart to I/R injury. ALDH2 activation can restore this aging-related myocardial ischemic intolerance.

## Introduction

Aged heart is more susceptible to ischemia/reperfusion (I/R) injury. The molecular mechanisms of aging related cardioprotection loss, however, are far from being elucidated. Sirtuin 1 (SIRT1), an NAD^+^-dependent protein deacetylase, has been proved to be an effective protector against age-related cardiovascular diseases. Recent human genetic studies also identified a role of SIRT1 in maintaining human health status with aging [1]. Activation of SIRT1 not only suppresses apoptosis but also balances oxidative stress in the heart [2], while absence of SIRT1 triggers chronic inflammation [3], cell cycle arrest [4] and early neonatal death [5]. SIRT1 is considered to be a potential interventional target for I/R injury management in the elderly.

Aging is characterized as progressive exacerbation of cells and organs due to accumulation of macromolecular and organelle damage. Recent evidences have revealed that endogenous reactive aldehydes (such as 4-hydroxynonenal, 4-HNE and malondialdehyde, MDA) could severely impair cardiac functions, which ultimately contributes to cardiac diseases [6]. Compared with reactive oxygen species (ROS), aldehyde is an enduring toxic agent by covalent modification of protein, such as carbonylation, leading to accumulation of damaged proteins in aged cells and organisms [7,8]. Therefore, it could be speculated that aldehyde toxicity accumulation may be involved in the aging related loss of cardioprotection. Several discoveries have confirmed that normal cells maintain a defensive detoxification capacity to prevent acute or chronic build-up of toxic aldehydes. ALDH2, an abundantly expressed protein in heart and brain, plays a major role in aldehyde detoxification [9]. Moreover, as a potent cardioprotective enzyme, ALDH2 has been reported to ameliorate cardiac toxicity of ethanol and reduce ischemic damage [10,11]. On the contrary, ALDH2 deficiency has also been considered to be responsible for the oxidative stress-related diseases, especially aging related cardiovascular diseases [12]. Within this context, reducing the ‘aldehydic overload’ by ALDH2 activation is a potential therapy for aging-related susceptibility to I/R injury.

In the present study, we report for the first time that cardiac SIRT1 was modified by aldehyde mediated carbonyl stress, which led to aging-related ischemic intolerance. Furthermore, we demonstrated pharmacological ALDH2 activation restored SIRT1 impairment. Our data suggested that forestalling SIRT1 carbonyl stress by ALDH2 activation would be an ideal target for protecting aged heart against I/R injury.

## Methods

The experiments were performed with adherence to the National Institutes of Health Guidelines for the Use of Laboratory Animals. Approval for this study was granted by the Animal Ethical Experimentation Committee of Fourth Military Medical University.

### Experimental protocol

Male C57BL/6 mice (4-6 and 22-24 mo) were purchased from animal center of Fourth Military Medical University. SIRT1 heterozygote KO (Sirt1^+/−^) mice (4-6 mo) were obtained from The Jackson Laboratory (Bar Harbor, ME). *Sirt1* ^+/−^ mice were backcrossed with C57BL/6 mice for 6 generations to obtain the C57BL/6 gene background. Age-matched (4-6 mo) wild-type (*Sirt1* ^+/+^) and heterozygous (*Sirt1* ^+/−^) littermate male mice were used in the study. Mice were maintained on a 12-h light-dark cycle in a controlled environment with water *ad libitum*.

### ALDH2 enzymatic activity

ALDH2 enzymatic activity was determined by measuring the conversion of NAD^+^ to NADH at absorbance of 340 nm, as described [13]. ALDH2 activity was measured at 25°C in 33 mmol/L sodium pyrophosphate containing 0.8 mmol/L NAD^+^, 15µmol/L propionaldehyde, and 0.1 ml protein extract (50 µg of protein). Propionaldehyde, the substrate of ALDH2, was oxidized in propionic acid, whereas NAD^+^ was reduced to NADH to estimate ALDH2 activity. NADH was determined by spectrophotometric absorbance at 340 nm. ALDH2 activity was expressed as nmol NADH/min per mg protein.

### SIRT1 activity assay

SIRT1 deacetylase activity was evaluated in crude nuclear extract from heart samples [14]. Trichostain A (0.2 mM; Sigma-Aldrich, St. Louis, MO, USA), components of Fluor de Lys SIRT1 Fluorescent Activity Assay/Drug Discovery Kit (Enzo Life Sciences, Farmingdale, NY, USA), including 100 µmol/L fluorogenic peptide encompassing residues 379 to 382 of p53 with lysine 382 being acetylated, and 170 µmol/L NAD^+^ at 37°C for 1 h, followed by incubation in developer for 15 min at room temperature according to the manufacturer’s instructions. Fluorescent intensity was measured using a Fluoroskan Ascent® microplate fluorometer (Thermo Electron Corp., Milford, MA, USA). No-enzyme and time 0 negative controls were generated by incubating developer II solution with 2 mmol/L NAM before mixing the substrates with or without samples. SIRT1 activity was calculated with the corrected arbitrary fluorescence units of the tested samples to no-enzyme control and expressed as fluorescent units relative to the control. The nuclear-cytoplasmic fraction of heart tissue was conducted using the NE-PER Nuclear and Cytoplasmic Extraction Reagents kit (Thermo, Fisher Scientific, Rockford, IL, USA) [14].

### Protein-HNE adducts determination

Protein-HNE adducts were determined in homogenates as fluorescence exhibited by interaction between the amino acid residues of protein and 4-HNE at 355/460 nm excitation/emission, respectively, and results were expressed in arbitrary units [15].

### Total protein and SIRT1 carbonylation assessment

Samples were resuspended in 10 mmol/L 2,4-dinitrophenylhydrazine (DNPH) solution for 30 min at room temperature before 20% trichloroacetic acid was added. Samples were centrifuged and the precipitate was resuspended in 6 mol/L guanidine solution. Maximum absorbance (360–390 nm) was read against appropriate blanks, and carbonyl content was calculated using the formula: absorption at 360 nm×45.45 nmol/protein content (mg) [11]. Additional studies were performed to detect covalent modification of SIRT1 by carbonylation. SIRT1 was immunoprecipitated using whole-cell extracts in accordance with published methods [16]. To determine the carbonylation of SIRT1, blots were probed first with anti-SIRT1 antibody. After stripping, membranes were equilibrated with 20% (v/v) methanol, 80% Tris-buffered saline for 5 min. Then they were incubated with 0.5 mmol/L 2,4-DNPH for 30 min at room temperature. The membranes were washed and then incubated overnight in anti-DNPH antibody (Abcam), as described previously [17].

### Cardiomyocyte isolation and cell viability assays

Adult mice ventricular myocytes were isolated by a standard enzymatic technique as described previously [11]. Cultured myocytes were subjected to hypoxia in an air-tight chamber under serum-free and no glucose culture Dulbecco’s Modified Eagle Medium (DMEM) continually gassing with 95% nitrogen with 5% CO_2,_ and then cells were taken out from chamber with serum-free DMEM to reoxygenation (room air with 5% CO_2_). Cardiomyocytes were pretreated with the 4-HNE (10 µmol/L, Sigma), Alda-1 (20 µmol/L, Calbiochem), SRT1720 (1 µmol/L, Cayman Chemical) or EX527 (10 µmol/L, Tocris Bioscience) for 1 hr at 37°C before 1 hr exposure to hypoxia followed by 1 hr reoxygenation. Generation of reactive oxygen species (ROS) was measured using chloromethyl-2',7'-dichlorodihydrofluorescein (CM-H2DCFDA) diacetate as previously described [18]. Cardiomyocyte viability was assayed by MTT as described previously [11]. The cardiomyocytes were plated in microtitre plate at a density of 3×10^5^ cells/mL. MTT was added to each well with a final concentration of 0.5 mg/mL, and the plates were incubated for another 2 h at 37°C. Formazan was quantified spectroscopically at 560 nm using a SpectraMaxw 190 spectrophotometer.

### Caspase-3 assay

Caspase-3 activity was determined according to the published method [13]. In brief, myocytes were lysed in 100 µl of ice-cold cell lysis buffer (50 mmol/l HEPES, 0.1% CHAPS, 1 mmol/l dithiothreitol, 0.1 mmol/l EDTA, 0.1% NP40). Following cell lysis, 70 µl of reaction buffer and 20 µl of caspase-3 colorimetric substrate (Ac-DEVD-p-nitroanilide) were added to cell lysate and incubated for 1 h at 37°C, during which time, caspase enzyme in the sample was allowed to cleave the chromophore pNA from its substrate molecule. Absorbency was detected at 405 nm, with caspase-3 activity being proportional to the color reaction.

### In vivo regional ischemia and experimental myocardial infarction

Mice were anaesthetized with 3% isoflurane, intubated, and ventilated with oxygen (Rodent Ventilator, Harvard Apparatus, Millis, MA, USA). The core temperature was maintained at 37°C with a heating pad. The surgical procedures used for LAD ligation were performed as described [19]. After left lateral thoracotomy, the left anterior descending artery (LAD) was occluded for 30 min with an 8-0 nylon suture and polyethylene tubing to prevent arterial injury, prior to 4 h reperfusion. ECG traces confirmed the ischemic hallmark of ST segment elevation during coronary occlusion (ADInstruments, Colorado Springs, CO, USA). At different time points, the left ventricle (LV) was isolated before freeze-clamping in liquid nitrogen. Freeze-clamped LV was stored at -80°C for further immunoblotting analysis. Myocardial infarct size was determined by means of a double-staining technique and a digital imaging system (infarct area/area-at-risk×100%) as described previously [19]. Non-necrotic tissue in the ischemic region was stained red with 2,3,5-triphenyltetrazolium chloride (TTC; 1% w/v), and the nonischemic region was stained blue with Evan’s blue (1% w/v). Hearts were fixed by 4% formalin overnight at 4°C, then sectioned into 1-mm slices, photographed using a Leica microscope (Leica Microsystems, Wetzlar, Germany), and analyzed using ImageJ software (U.S. National Institutes of Health, Bethesda, MD, USA). The myocardial infarct size was calculated as the extent of myocardial necrosis to the ischemic area at risk (AAR). Continuous infusion of Alda-1 (16 µg/g, Tocris Bioscience) or vehicle was administered via tail vein 2 hr before ischemia, as described previously [20]. Blood samples for creatine kinase (CK) activity measurement were collected 4 h after reperfusion from mice subjected to I/R and determined spectrophotometrically at 340 nm as described previously [21].

### Western blotting analysis

Immunoblots were performed as previously described [22]. Rabbit polyclonal antibody against SIRT1 was purchased from Santa Cruz Biotechnology (Santa Cruz, CA, USA). Antibodies against p16, tubulin, GAPDH, TATA-binding protein (TBP), and horseradish peroxidase linked secondary antibodies were purchased from Cell Signaling.

### Mouse heart perfusion

Mice were given an injection of heparin (100 U i.p.) 10 min prior to pentobarbital sodium (60 mg/kg i.p.)-induced euthanasia. The hearts were quickly excised and retrogradely perfused (4 ml/min) on a Heart Perfusion System (Radnoti Glass Technology, Monrovia, CA, USA) with 95% O_2_ and 5% CO_2_ equilibrated Krebs-Henseleit buffer (118 mM NaCl, 4.75 mM KCl, 1.2 mM KH_2_PO_4_, 1.2 mM MgSO_4_, 25 mM NaHCO_3_, 1.4 mM CaCl_2_ containing) containing 7 mmol/l glucose, 0.4 mmol/l oleate, 1% BSA, and a low fasting concentration of insulin (10 µU/ml). For generating the ex vivo ischemic model, buffer flow was cut off for 20 min after 30 min of preperfusion of buffer containing Alda-1 (20 µmol/L in 0.01% DMSO) or vehicle (0.01% DMSO). The hearts were then reperfused with the same rate of buffer flow during reperfusion. The LabChart6 software (ADInstruments) was used to monitor the heart rate and left ventricular developed pressure, as described previously [19]

### Statistical analysis

All values were presented as means±SEM. Differences were compared by ANOVA followed by Bonferroni correction for post hoc t test, where appropriate. Probabilities of <0.05 were considered to be statistically significant. All of the statistical tests were performed with the GraphPad Prism software version 5.0 (GraphPad Software, San Diego, CA).

## Results

### Impaired ALDH2 and SIRT1 activity in aged heart

**Figure 1:**
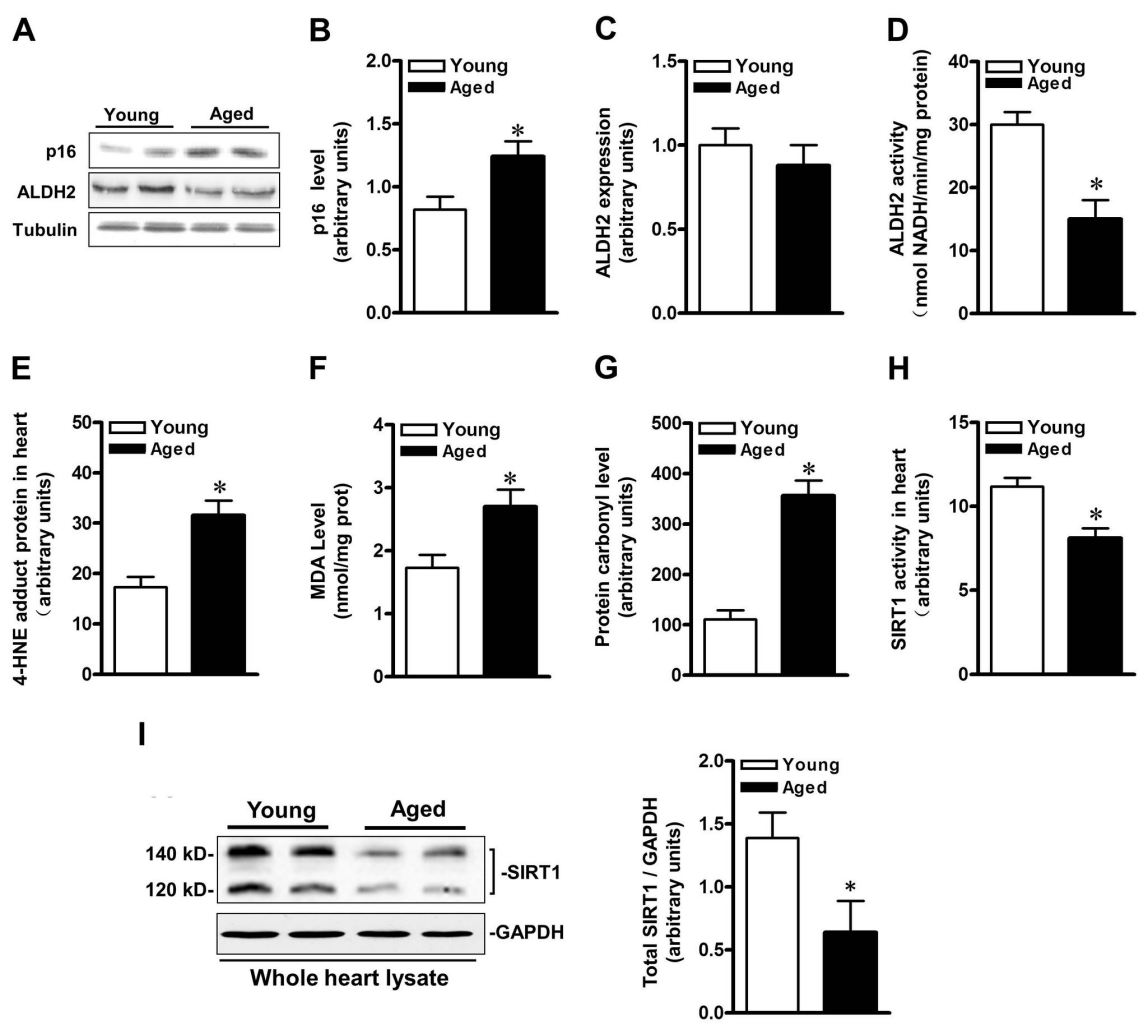
Impaired ALDH2 and SIRT1 activity in aged hearts. (**A**) Representative gel blots depicting (**B**) p16 and (**C**) ALDH2 protein expression in young and aged hearts. Quantification showing (**D**) decreased ALDH2 activity; (**E**) increased 4-HNE protein adducts formed; (**F**) increased MDA levels; (**G**) increased protein carbonyl level; (**H**) decreased cardiac SIRT1 activity ; (**I**) decreased cardiac SIRT1 protein expression levels in aged heart. (n=8 per group. *P<0.05 vs. young).

The myocardial senescence marker, ALDH2 protein expression and activity in young and aged C57BL/6 mice were assayed. Expression of p16, a marker of senescence, was significantly increased in the aged heart (Figure 1A,B). Aged heart exhibited a decline trend in ALDH2 protein expression but with no significant difference. However, myocardial ALDH2 activity decreased in aged hearts compared with that in their younger counterparts (Figure 1C,D; *P*<0.05). Meanwhile, the cardiac 4-HNE-protein adducts, MDA level and carbonylated proteins were markedly higher in aged group than those in young groups (Figure 1E–G; both *P*<0.05). Furthermore, aged hearts also demonstrated a 27% drop in SIRT1 activity (Figure 1H; P < 0.05). Both nuclear (140 kDa) and cytoplasmic (120 kDa) SIRT1 protein levels are decreased in aged hearts (both *P*<0.05; Figure 1I). Although SIRT1 are down-regulated in aging mice heart, these results still indicate that aging impairs cardiac ALDH2 activity and increases aldehyde/carbonyl stress, possibly leading to decreased SIRT1 activity.

### Activation of ALDH2 prevented SIRT1 against aldehyde-induced carbonyl modification

**Figure 2:**
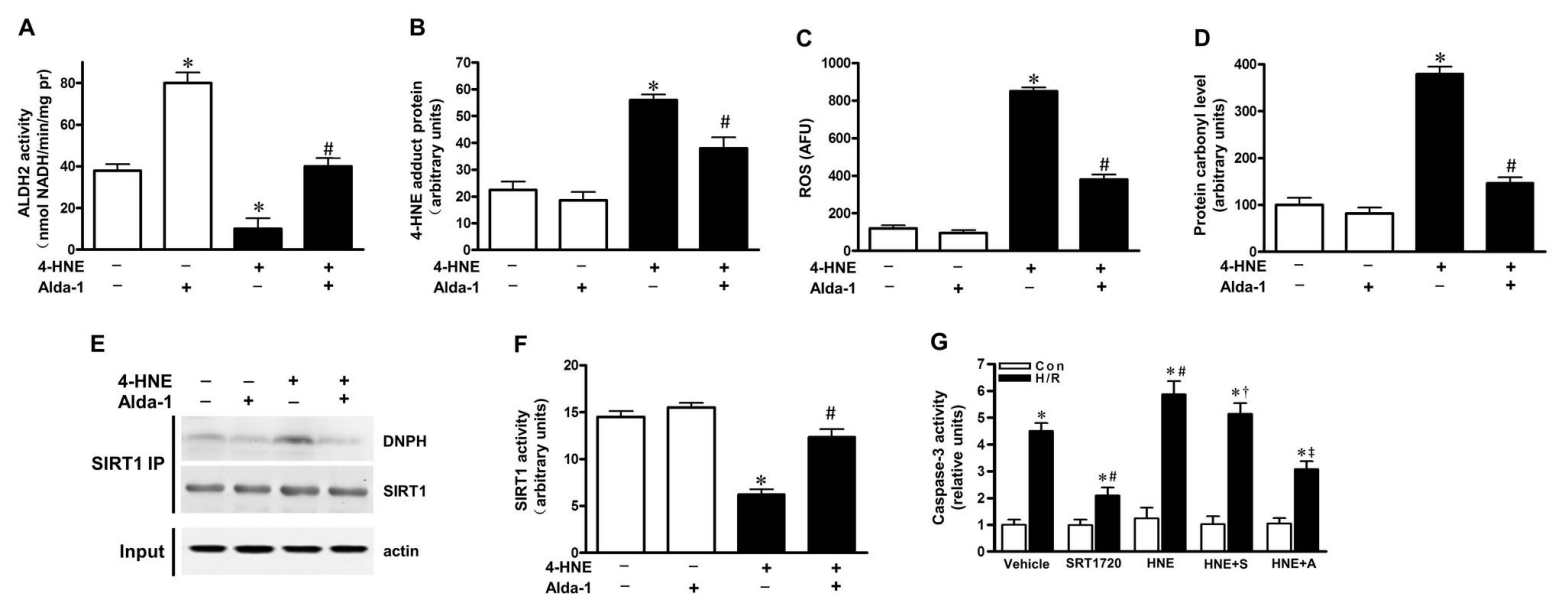
Alda-1 treatment prevented the harmful effect of 4-HNE on cardiomyocytes. Isolated cardiomyocytes with or without Alda-1 treatment (20 µmol/L) were incubated with 4-HNE (10 µmol/L) or vehicle for 1 hr. (**A**) ALDH2 activity; (**B**) 4-HNE adduct protein; (**C**) ROS generation; (**D**) protein carbonyl formation and (**F**) cardiac SIRT1 activity were assessed by quantificational detection (n=8 per group. *P<0.05 vs. vehicle control, #*P* <0.05 vs. 4-HNE alone). (**E**) Representative immunoprecipitation (IP) picture was used to confirm carbonylation of SIRT1. (**G**) Cardiomyocytes were pretreated for 1 h with vehicle, SRT1720 (1 µmol/L), 4-HNE (10 µmol/L) or 4-HNE plus SRT1720 or Alda-1(20 µmol/L), and then with or without 1 hr of hypoxia and 1 hr reoxygenation (H/R). Quantification showing cardiomyocytes caspase-3 activity. (n=8 per group. *P<0.05 vs vehicle control, #*P* <0.05 vs. vehicle H/R, † *P* <0.05 vs SRT1720 H/R, ‡ *P* <0.05 vs. HNE H/R).

As 4-HNE has been proved to be a highly reactive carbonyl compound and 4-HNE adduct formation in the myocardium may lead to the inhibition of key metabolic enzymes [6], we then examined whether excessive aldehyde stress may be responsible for the SIRT1 inactivity in cardiomyocytes. Isolated cardiomyocytes exposed to exogenous 4-HNE (10 µmol/L)-induced aldehyde stress with or without ALDH2 activator Alda-1 (20 µmol/L) treatment. It was noted that 4-HNE significantly reduced ALDH2 and SIRT1 activities, increased 4-HNE adducts formation, ROS generation, and carbonylated proteins in cardiomyocytes (Figure 2A–D; all *P*<0.05). However, Alda-1 treatment rescued cardiomyocytes from ROS attack, inhibited protein carbonyl formation, and restored ALDH2 activity (Figure 2A–D; all *P*<0.05). Moreover, SIRT1 immunoprecipitation and immunoblotting findings indicated that 4-HNE exposure led to increased carbonylated SIRT1 and markedly decreased SIRT1 activity, but Alda-1 treatment significantly reduced the level of carbonylated SIRT1 and restored the SIRT1 activity (Figure 2E,F; *P*<0.05). These results illustrated that cardiac SIRT1 was the carbonylation target by aldehyde stress which consequently resulted in SIRT1 inactivation, while Alda-1 treatment activates SIRT1 in cardiomyocytes by preventing aldehyde-induced carbonyl modification on SIRT1.

### Carbonyl stress increased susceptibility of cardiomyocytes to hypoxia and reoxygenation (H/R) injury

To further explore the biological consequence of 4-HNE accumulation in cardiomyocytes with H/R insult and the relationship among ALDH2, aldehyde/carbonyl stress and SIRT1 activity, isolated cardiomyocytes were incubated with 4-HNE or vehicle for 1 h prior to H/R treatment. The results demonstrated that 4-HNE exposure aggravated H/R-induced myocardial caspase-3 activity (Figure 2G; P < 0.05). Moreover, SIRT1 activator SRT1720 treatment inhibited H/R induced caspase-3 activity in isolated cardiomyocytes, while 4-HNE exposure significantly limited the protective effect of SRT1720 on H/R-induced cardiomyocyte injury (Figure 2G; P < 0.05). However, pretreated with Alda-1 blocked the harmful effect of 4-HNE on H/R-induced cardiomyocyte injury. When coupled with the SIRT1 carbonyl modification data established that aldehydes exposure resulted in serious cardiomyocyte toxicity, including accentuated SIRT1 carbonylation and impaired myocardial tolerance to H/R injury, and ALDH2 activation protected myocardium against H/R injury via reducing aldehydic cytotoxic.

**Figure 3:**
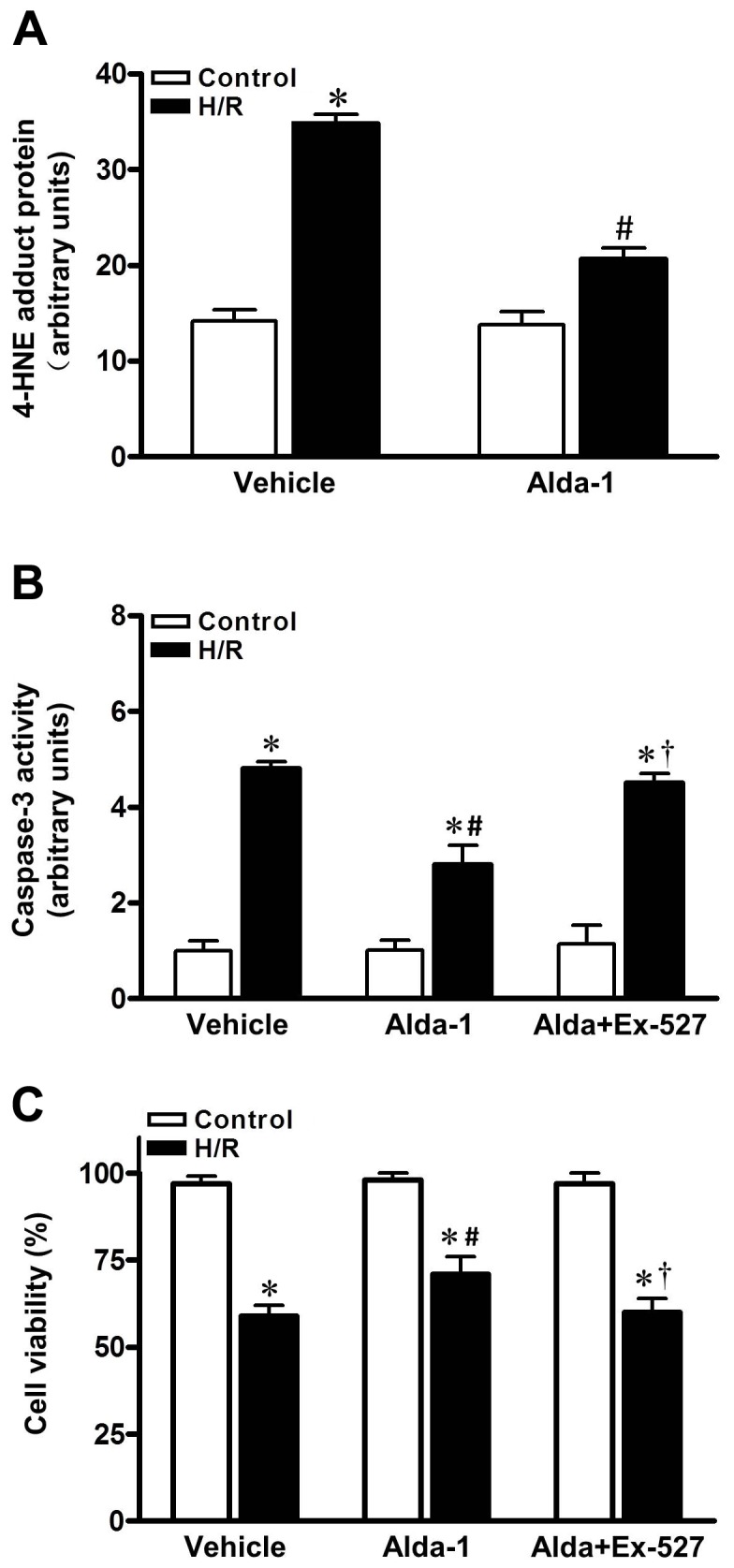
Alda-1 treatment protected cardiomyocytes against H/R injury. Cardiomyocytes were pretreated for 1 h with vehicle, Alda-1(20 µmol/L) or Alda-1 plus Ex-527 (10 µmol/L), and then with or without 1 hr of hypoxia and 1 hr reoxygenation (H/R). Quantification showing (**A**) endogenous 4-HNE adduct protein, (**B**) cardiomyocytes caspase-3 activity, (**C**) cardiomyocyte death. (n=8 per group. *P<0.05 vs. vehicle control; #*P* <0.05 vs. vehicle H/R, † *P* <0.05 vs. Alda-1 H/R).

Our data depicted that exogenous 4-HNE content carbonylated cardiac SIRT1 and blunted its activity, which contributed to myocardial intolerance to H/R injury. Interestingly, endogenous 4-HNE was also produced by H/R insult. As illustrated in Figure 3A, a significant elevation of 4-HNE adducts formation was detected in cardiomyocyte with H/R, which was markedly depressed by Alda-1 treatment. As a result, increased myocardial caspase-3 activity and cardiomyocyte death by H/R insult were also significantly depressed by Alda-1 treatment (Figure 3B,C; *P*<0.05). However, SIRT1 inhibitor Ex-527 (10 µmol/L) blocked the protective effect of Alda-1 on H/R induced cardiomyocyte injury. These results indicated that SIRT1 activity is necessary for the cardioprotective effect of ALDH2 that detoxifies endogenous or exogenous myocardial aldehyde stress during H/R.

### Alda-1 activated SIRT1 via downregulation of endogenous 4-HNE in aged heart with I/R attack

**Figure 4:**
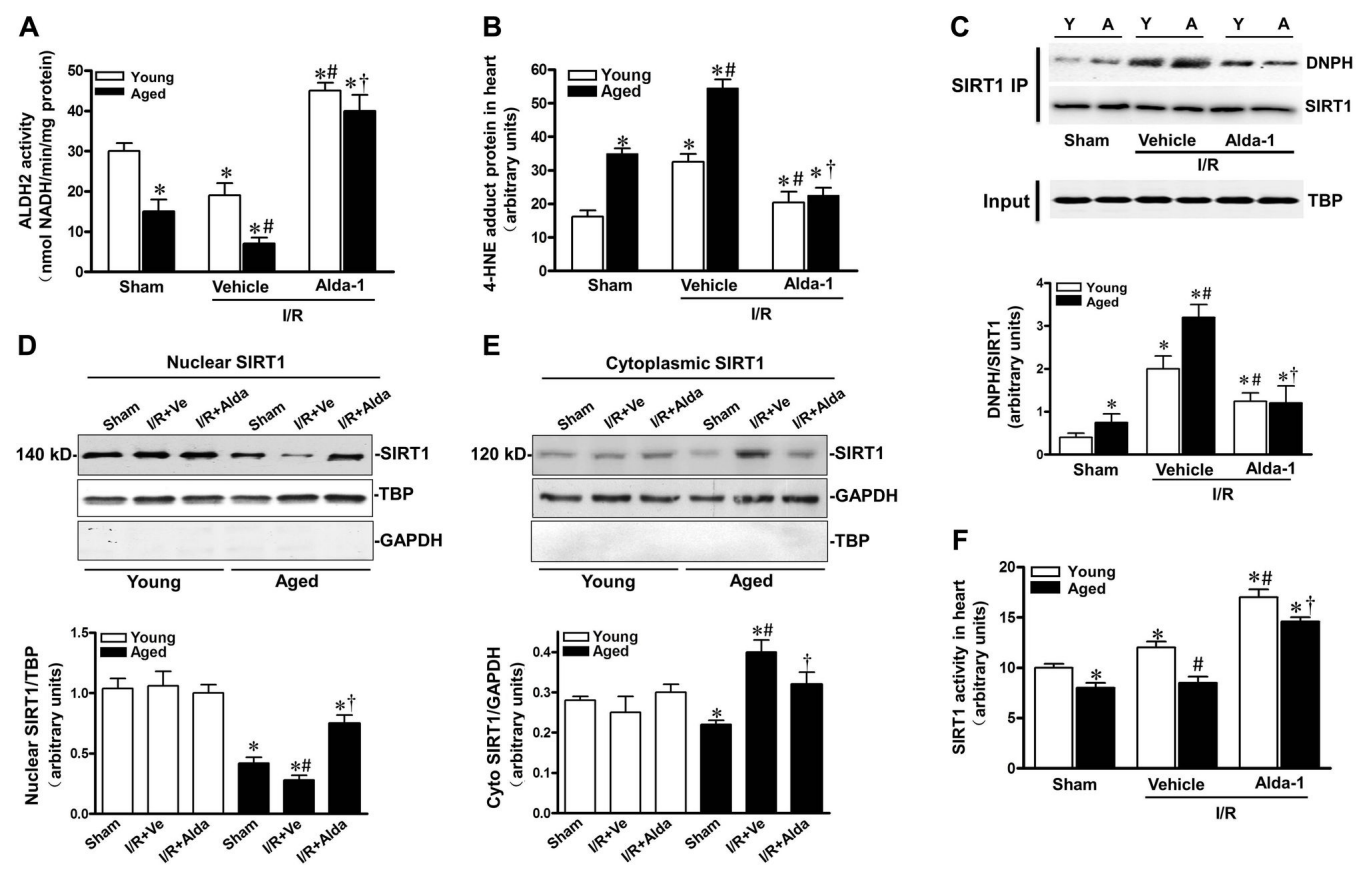
Alda-1 activated SIRT1 in aged hearts during I/R. Young and aged C57BL/6 mice were subjected to 30-minute coronary artery ligation followed by 1-hour reperfusion *in vivo*, Alda-1 (16 µg/g) or vehicle was administered via tail vein 2 hr before ischemia. (**A**) ALDH2 activity and (**B**) HNE protein adducts formation were assessed. (**C**) Nuclear extracts from young and aged hearts were subjected to immunoprecipitation (IP) with SIRT1 antibody. The IP products were further analyzed by immunoblotting; anti-DNPH and anti-SIRT1 antibodies were reciprocally used to confirm carbonylation of cardiac SIRT1. Anti-TBP was used as a input ; bar graphs show relative levels of carbonylation of cardiac SIRT1 in young and aged hearts. (**D**) Nuclear and (**E**) cytoplasmic extracts from young and aged hearts were analyzed by immunoblotting. TBP and GAPDH were detected as nuclear and cytoplasmic loading control, respectively. Bar graphs show relative levels of nuclear SIRT1 and cytoplasmic SIRT1 from young and aged hearts. (**F**) Nuclear fractions of young and aged LVs were subjected to the SIRT1 activity assay. (n=6-8 per group. *P<0.05 vs. young sham; #*P* <0.05 vs. young I/R vehicle; † *P* <0.05 vs. Aging I/R vehicle).

4-HNE was reported to be produced during I/R insults *in vivo* [11]. To further explore *in vivo* role of ALDH2 in regulation of cardiac SIRT1 activity during I/R, young and aged mice were subjected to myocardial I/R injury, and the relationship between these factors were evaluated. After 30-minute coronary artery ligation and 1-hour reperfusion, I/R markedly inhibited ALDH2 activity, which was more worsened in aged hearts than that in young ones as evidenced by 63% decrease of ALDH2 activity in aged hearts (Figure 4A; P < 0.05). In addition, I/R induced 4-HNE-protein adducts in aged hearts was 1.7-fold higher than that seen in I/R young hearts (Figure 4B; P < 0.05). However, Alda-1 (16 µg/g) given 2 hr prior to ischemia significantly elevated myocardium ALDH2 activity and inhibited 4-HNE-protein adduct formation in aged hearts (Figure 4 A,B; both *P*<0.05). Moreover, aged hearts with I/R attack showed more SIRT1 carbonylated modification versus young groups (Figure 4C), whereas ALDH2 activation by Alda-1 led to a significant reduction of SIRT1 carbonylation in aged hearts (*P*<0.05).

Nucleocytoplasmic shuttling plays a critical role in regulating SIRT1 activity. To further determine whether aging altered the rate of SIRT1 nuclear-to-cytosolic shuttling in response to I/R in the heart, nuclear (140-kDa) and cytoplasmic (120-kDa) SIRT1 were detected in young and aged hearts. There are no significant differences of nuclear and cytoplasmic SIRT1 among young groups, and aging markedly decreased nuclear SIRT1 but with a light downregulation of cytoplasmic SIRT1 (Figure 4D, E; *P*<0.05). Moreover, I/R elicited a notable reduction of nuclear SIRT1 but with a sharp increase of cytoplasmic SIRT1 in aged hearts (Figure 4D, E; *P*<0.05). This was accompanied with upregualtion of cardiac SIRT1 activity in young mice subjected to I/R surgery, but downregulation of SIRT1 activities in aged mice with or without I/R (Figure 4F; P < 0.05). These results suggest that aging impaired SIRT1 nucleus shuttling and I/R further this process, contributing to decreased SIRT1 activity. Since it was observed in our experiment that ALDH2 activation upregulated SIRT1 activity, we further explored the role of ALDH2 activation in regulation of SIRT1 nuclear shuttling and activity. Evidently, Alda-1 treatment resulted in an increase of nuclear SIRT1 and a decrease of cytoplasmic SIRT1 in response to I/R in aged heart (Figure 4D, E; *P*<0.05), resulting in improvement of SIRT1 activity (Figure 4F; P < 0.05). These results indicated that aging impaired cardiac SIRT1 nuclear translocation and activity, which could be rescued by ALDH2 activation. Combined with the *in vitro* findings, it could be inferred that SIRT1 carbonyl modification is a factor involved in the vulnerability of aged heart to I/R stress, which could be prevented by ALDH2 activation.

### Alda-1 treatment improved the tolerance of aged heart to I/R insult

**Figure 5:**
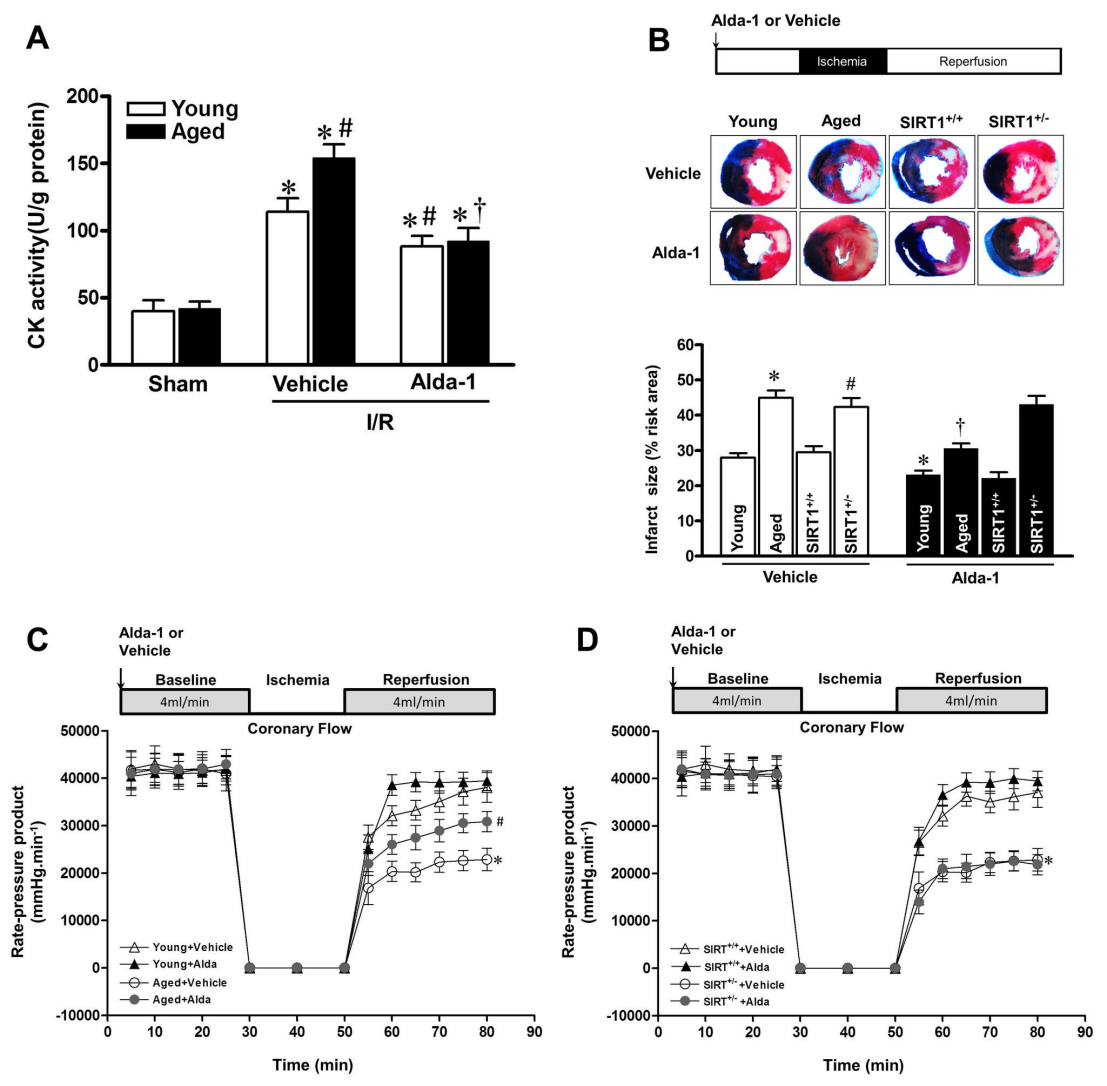
Alda-1 treatment improved the tolerance of aged hearts during I/R. Young and aged C57BL/6 mice were subjected to 30-minute coronary artery ligation followed by 4-hour reperfusion, Alda-1 (16 µg/g) or vehicle was administered via tail vein 2 hr before ischemia. (**A**) Creatine kinase (CK) activity measurement were collected 4 h after reperfusion from young and aged I/R mice. (**B**) Young and aged C57BL/6 mice, Sirt1^+/−^ heterozygous knockout mice, and Sirt1^+/+^ wild-type littermate mice (C57BL/6 background) were subjected to 30-minute coronary artery ligation followed by 4-hour reperfusion, Alda-1 (16 µg/g) or vehicle was administered via tail vein 2 hr before ischemia. Representative pictures and quantification of ratio of infarct size to total myocardium. (n=6-8 per group. *P<0.05 vs. young vehicle; #*P* <0.05 vs. Sirt1^+/+^ wild-type vehicle; † *P* <0.05 vs. Aging vehicle). (**C**) Isolated hearts from young and aged C57BL/6 mice or (**D**) Sirt1^+/−^ heterozygous knockout mice and Sirt1^+/+^ wild-type littermate mice (C57BL/6 background) were subjected to *ex vivo* heart perfusion (Langendorff). Heart rate × left ventricular developed pressure was determined during baseline perfusion, global ischemia, and postischemic reperfusion with vehicle or Alda-1(20 µmol/L) administration. (n=6-8 per group. *P<0.05 vs. young or Sirt1^+/+^ wild-type vehicle; #*P* <0.05 vs. aged vehicle).

To characterize the role of ALDH2 induced SIRT1 activation in cardioprotection against I/R injury in aged hearts, the extent of myocardial injury evaluated after 30 min *in vivo* regional ischemia followed by 4 h reperfusion. Creatine kinase activity, a direct index of cardiomyocytes damage, was markedly elevated in I/R operated aged hearts, which was prevented by ALDH2 activation as evidenced by 40% drop of creatine kinase activity after Alda-1 treatment (Figure 5A). To further confirm that the ALDH2 activation elicited cardioprotection in aged heart is dependent upon SIRT1, SIRT1 deficient heterozygous (Sirt1^+/−^) mice and wild type littermates were subjected to the identical I/R conditions. The results showed that the infarct size of aged C57BL/6 mice was 1.8-fold larger than that of young mice (Figure 5B; P < 0.05). Sirt1^+/−^ showed a comparable infarct size with aged hearts (Figure 5B), while wild-type littermates had no apparent discrepancy in myocardial infarction compared to young hearts. Alda-1 (16 µg/g i.v.) given 2 hr prior to ischemia significantly reduced infarct size by 32% in aged mice, but not in Sirt1^+/−^ mice (Figure 5B). Moreover, *ex vivo* heart perfusion showed that postischemic contractile function was impaired in aged vs. young hearts (Figure 5C; P < 0.05). Intriguingly, Alda-1 treatment improved postischemic contractile function recovery in aged heart but not Sirt1^+/−^ heart (Figure 5D). These results indicated that SIRT1 deficiency disenabled the cardioprotective effect of ALDH2 against I/R injury in aged heart, implying a SIRT1 dependent manner of ALDH2 action.

## Discussion

Intrinsic aging enhances the cardiac susceptibility to I/R injury [19]. It is generally agreed that there is a correlation between aging induced myocardial dysfunction and the accumulation of damaged proteins [23]. Reactive aldehydes induce covalent carbonyl modification of protein by “carbonyl stress”, which can account for protein inactivation and excessive accumulation of damaged proteins in the cell [8]. We and others have shown that ALDH2 exerts detoxification against toxic aldehyde [6] and reduces cardiac damage caused by ischemic insult [11,20]. But whether pharmacological ALDH2 activation would diminish aging-related ischemic vulnerability remains incompletely understood. Furthermore, SIRT1 has been proved to be beneficial against age-related diseases. However, it is still unclear whether the *in vivo* cardioprotective effect of ALDH2 activation is dependent on SIRT1 or not. The findings from our current study revealed that *in vivo* and *in vitro* aldehyde/carbonyl stress led to increased carbonylation on cardiac SIRT1 resulting in its activity impairment, which contributed to aging-related myocardial susceptibility to I/R injury. However, ALDH2 activator Alda-1 could protect aged heart against I/R insult via improving cardiac SIRT1 activity by decreasing carbonyl stress and promoting nucleocytoplasmic shuttling of SIRT1. These findings provided convincing proof that pharmacological ALDH2 activation could be a novel impactful therapeutic strategy for ischemia heart disease, particularly for elders.

ALDH2 is a tetrameric enzyme and plays a key role in the metabolism of acetaldehyde and other toxic aldehydes. Studies to date have demonstrated that ALDH2 alleviates myocardial injury induced by acetaldehyde, alcohol and ischemic attacks [10,13]. While ALDH2 KO exacerbated cardiac I/R or H/R injury [11,24]. In addition, epidemiological studies revealed higher risks of MI [25], hypertension [26], osteoporosis [27] and diabetes [28] in humans carrying an inactivating mutation in ALDH2, especially in East Asian countries. These findings suggest that declined in ALDH2 activity is associated with serious clinical consequences and may contribute to stress intolerance. Nonetheless, little information is available with regard to the effect of ALDH2 on aging-related cardiac ischemic vulnerability. Data from our present study indicated that aged heart exhibited reduced cardiac activity of ALDH2. Alda-1(N-(1,3-benzodioxol-5-ylmethyl)-2,6-dichlorobenzamide, MW=324) is a recently identified allosteric activator of ALDH2. Alda-1 increases productive substrate-enzyme interaction and protects the enzyme from substrate-induced inactivation. Further, crystallographic studies of ALDH2-Alda-1 complexes also show that Alda-1 acts as a chemical chaperone by stabilizing the structurally impaired enzyme at the tetrameric interface as well as within the catalytic tunnel, leading to catalytic recovery [29]. Mochly-Rosen et al [20] recently reported that *in vivo* sustained treatment with Alda-1 abrogated nitroglycerin-induced ALDH2 inactivation. Previous finding also indicated that Alda-1 increased both wild-type ALDH2 and ALDH2*2 mutant activity, and reduced infarct size by 60% [10]. In the present study, Alda-1 treatment was chosen to activate ALDH2 *in vivo* and *in vitro*, which significantly improved the anti-stress ability of aged heart against I/R injury, as evidenced by reduced plasma creatine kinase activity, cardiomyocyte death and infarct size. These data convincingly supported the notion that ALDH2 is a potential target for ameliorating the intolerance of aged heart to ischemic insults.

It has been well established that ALDH2 activation exerts cardioprotective effect on aged heart, but the detailed molecular mechanism remains unknown. SIRT1 has long been presumed to be an anti-aging protein, and the beneficial effects of SIRT1 against aging related diseases have been demonstrated by many studies [30]. SIRT1 also helps mediate myocardial response to stress. Although it has been demonstrated impaired cardiac SIRT1 activity played a critical role in increased susceptibility of aged hearts to I/R injury [31], whether SIRT1 modulation is involved in the cadioprotection of ALDH2 in senescence heart remains unknown. Our finding revealed a significant decline in SIRT1 activity in the aged heart, in accordance with previous findings that cardiac SIRT1 activity is declined in senescence [32]. Owing to Sirt1^−/−^ mice are not viable in inbred strain backgrounds and show pleiotropic phenotypes in outcrossed strains, including small size, developmental defects and sterility [33], we used global Sirt1 knockout in SIRT1 deficient heterozygous (Sirt1^+/−^) mice. In addition, we demonstrated that ALDH2 activation by Alda-1 upregulated SIRT1 activity, consequently alleviated H/R or I/R injury in cardiomyocytes exposed to 4-HNE and aged heart, but not in Sirt1^+/−^ mice. Based on these findings, it was proved that SIRT1 deficiency would impair ALDH2 activation induced cardioprotection against I/R injury in aging. Namely, ALDH2 offered beneficial effects against aging-related myocardial ischemic intolerance via a sirtuin-dependent manner. To further explore the mechanism of aging related SIRT1 activity changes, we observed nuclear SIRT1 protein levels, a marker for active SIRT1 [31], in young and aged mice hearts. The result showed that aged heart exhibited lower nuclear SIRT1 level compared with those in young hearts, which was further downregulated under I/R insult. Interestingly, ALDH2 activation activated SIRT1 in aged heart by promoting its nuclear shuttling in response to I/R. These results provided a direct proof that in addition to reduced nuclear SIRT1 localization, the aged heart is incapable of mounting a robust SIRT1 response to ischemic stress, which could be rescued by ALDH2 activation. SIRT1 nucleocytoplasmic shuttling is regulated by post-translational modification such as sumoylation [31]. Some evidences exist that aging related reactive oxygen species increase may impair the activities of SUMO-1 and desumoylase in the heart, which could lead to degradation and impaired nucleocytoplasmic shuttling of SIRT1 in the aged heart [31]. Our data provided new insight that aging related excessive carbonyl stress is another important factor which is responsible for impaired nucleocytoplasmic shuttling of SIRT1 via posttranslational modification on SIRT1.

The question remained what was the possible reason for SIRT1 activity reduction in elderly heart. In this study, we have observed that SIRT1 was down-regulated in aging mice heart. SIRT1 expression is an important index of its diverse cellular functions; however, its expression cannot be used as the sole indicator of activity. Oxidants are known to cause the peroxidation of bioactive molecules. Carbonylation of proteins results from reactive aldehydes reacting with cysteine, histidine, and lysine residues by Michael addition [8], which leads to protein inactivation, degradation, and/or accumulation [6]. 4-HNE, a lipid peroxided of reactive aldehydes, reacts with biomolecules through catalyzing highly electrophilic carbonyl formation to generate various adducts, which ultimately results in protein inactivation [34]. Our previous studies have demonstrated that 4-HNE directly inhibited myocardial contractility [11]. In this study, exogenous 4-HNE induced carbonylated SIRT1 was investigated by immunoprecipitation and anti-SIRT1 together with anti-DNPH blotting [16]. Our results demonstrated that 4-HNE exposure lead to increased carbonylated SIRT1, while Alda-1 treatment activates SIRT1 in the aged cardiomyocytes by preventing aldehyde-induced carbonyl modification on SIRT1. Pretreatment with Alda-1 blocked the harmful effect of 4-HNE on H/R-induced cardiomyocyte injury. These results indicated that cardiac SIRT1 is the carbonylation target for aldehyde stress which impairs cardiac SIRT1 activity and ultimately mediates increased myocardial susceptibility to I/R injury.

Aldehydic accumulation accounts for several pathological conditions associated with malignant stress [11]. In addition, ALDH2 substrates such as 4-HNE and other inhibitors also impair its activity. There are abundant evidences indicating detrimental role of the endogenous 4-HNE product in cardiac injury during ischemia and reperfusion *in vivo*. Moreover, 4-HNE accumulation has also been implicated in the development of aging-related system impairment [34,35]. In this study, we found that accumulations of aldehydes, especially 4-HNE, correlated well with decreased ALDH2 activity in aged heart, and 4-HNE treatment elicited more myocardial ROS release and ALDH2 inactivation. Besides this, elevated 4-HNE protein adducts remarkably increased after I/R insult in aged heart. However, the activation of ALDH2 inhibited the aging and I/R- induced accumulation of 4-HNE adducts. These findings indicated that a reciprocal feedback loop between ALDH2 and aldehydic homeostasis, and oxidative stress, while ALDH2 inactivation and reactive aldehydes accumulation set up a vicious circle during aging, causing chemical modification of bioactive molecules. Given extravagant aging-related myocardial ‘aldehydic load’ was responsible for intolerance of the senescent heart to I/R insult, pharmacological ALDH2 activation provides an effective solution for ameliorating this aging-related unbalance, which providing a strong experimental proof for clinical application of ALDH2 agonist in patient with MI and other ALDH2-related diseases, especially for the elder.

In conclusion, our results demonstrated that decreased ALDH2 activity in aged heart accounted for myocardial aldehydic overload, which induced carbonyl modifications on SIRT1, and ALDH2 activitor Alda-1 provided aged heart with an effective protection against I/R injury in a SIRT1-dependent manner. A new link between aldehyde toxicity induced carbonyl stress and aging-related myocardial ischemic vulnerability was established.
